# 

**Published:** 1892-10-22

**Authors:** 


### CONTENTS.

| | PAGE |
|---|---|
| **The Wisdom of the Moderns** | 49 |
| **Passing Topics.**—To Will and to Do | 50 |
| **Diet in Disease.** By Mrs. Ernest Hart. IV.—Stimulants (*continued*): The Coca of Peru—Coca Wine | 51 |
| **Editor's Letter-Box.**—The Public School Boy's Body | 52 |
| **Medical History from the Earliest Times.** By E. T. Withington, M.A., M.B., Oxon. XXVIII.—Arabic Medicine—(1) The Translators | 53 |
| **Everybody's Page** | 54 |
| **Annotations.**—A Home of Peace for the Dying—Drunk or Dying? —Experiments on Human Subjects | 55 |
| **Notes and News** | 56 |
| **Books Received** | 60 |
| **The Student of Medicine.**—Notes from the Schools—Appointments—Vacancies | |
| **The Hospital Clinic—** | |
| Royal Free Hospital.—Treatment of Burns and Scalds | 57 |
| St. Bartholomew's Hospital.—The Treatment of Typhoid Fever | 58 |
| The Queen's Hospital, Birmingham.—The Treatment of Sprains of the Ankle | 59 |
| New Drugs, Appliances, and Things Medical.—The "Carboline" Disinfectant | 60 |
| **The Institutional Workshop—** | |
| Within the Hospitals.—Morningside Asylum, Edinburgh. By Our Special Commissioner | 61 |
| Hospital Construction.—The "W. H. Smith" Isolation Hospital, Henley-on-Thames | 62 |
| Hospital Opinion.—The Aberdeen Hospital for Sick Children—The Central London Throat and Ear Hospital | 64 |

### EXTRA SUPPLEMENT.—THE NURSING MIRROR.

| | |
|---|---|
| **En Passant.**—Tottenham Temporary Fever Hospital—Bath Royal United Hospital Private Nurses—The Nurses' Co-operation—Bolton District Nursing Association—Anonymous Correspondents—Bangles at Bedsides—Short Items | xix |
| **Lectures for Asylum Attendants.** By William Harding, M.B.—V. Food (*continued*) | xx |
| **Christmas Competitions** | xx |
| **From a Private Patient's Point of View** | xxi |
| **Appointments** | xxi |
| **Nurses' Bookshelf.**—"The Art of Massage" | xxii |
| **For Reading to the Sick.**—Growth | xxii |
| **Everybody's Opinion.**—Degrees of Training—Metropolitan District Schools—Signing Agreements—Royal Commission, Chicago Exhibition, 1893—"Wanted, a Resident Yardsman" | xxiii |
| **Presentations** | xxiii |
| **"The Result of a Chill."**—III. Some Notable Pictures | xxiv |
| **Notes and Queries** | xxiv |
| **Wants and Work rs** | xxiv |

